# Rigidity, dyskinesia and other atypical overdose presentations observed at a supervised injection site, Vancouver, Canada

**DOI:** 10.1186/s12954-018-0271-5

**Published:** 2018-12-22

**Authors:** Mai-Lei Woo Kinshella, Tim Gauthier, Mark Lysyshyn

**Affiliations:** 1 Vancouver Coastal Health, Vancouver, Canada; 2 School of Population and Public Health, University of British Columbia, Vancouver, Canada; 3 Office of the Medical Health Officer, 5th Floor, 132 West Esplanade, North Vancouver, Canada

**Keywords:** Opioid overdose, Fentanyl, Atypical overdose presentations, Rigidity, Dyskinesia, Supervised consumption sites

## Abstract

**Objective:**

In midst of the overdose crisis, the clinical features of opioid overdoses seem to be changing. Understanding of the adverse effects of synthetic opioids such as fentanyl is currently limited to clinical settings. Insite, a supervised injection site in Vancouver, Canada, provides an opportunity to better understand illicit drug overdose presentations.

**Methods:**

A review of clinical records at Insite for October 2016 to April 2017 was undertaken to quantify atypical overdose presentations. Overdose reports were reviewed for the number of atypical opioid overdose presentations, temporal trends over the study period, concurrent symptoms, and interventions employed by staff.

**Results:**

Insite staff responded to 1581 overdoses during the study period, including 497 (31.4%) that did not fit a typical presentation for opioid overdoses. Of these, 485 fit into five categories of atypical features: muscle rigidity, dyskinesia, slow or irregular heart rate, confusion, and anisocoria. Muscle rigidity was the most common atypical presentation, observed in 240 (15.2%) of the overdose cases, followed by dyskinesia, observed in 150 (9.2%). Slow or irregular heart rate was observed in 69 (4.4%) cases, confusion in 24 (1.5%), and anisocoria in 2 (0.1%) of overall overdose cases.

**Discussion:**

The similarity of atypical overdose cases at Insite with anesthesiology case reports supports the understanding that the illicit drug supply is contaminated by fentanyl and other synthetic opioids. Atypical overdose presentations can affect clinical overdose response. The experience at Insite highlights the potential for supervised consumption sites to be innovative spaces for community learning and knowledge translation.

## Background

Insite, North America’s first legal supervised injection site, is located in the Downtown Eastside neighborhood of Vancouver, Canada. Supervised consumption sites hold exemptions from drug laws so that people who use drugs can use their own drugs under supervision of nurses and staff. In addition to preventing overdose deaths and providing a safe space, the facility also facilitates connections to addiction treatment and medical care. In the winter of 2016 and the spring of 2017, nurses and harm reduction workers at Insite were faced with a double challenge. First, drug overdoses were on the rise as the illicit drug supply was increasingly being contaminated with fentanyl [1]. A study in the Downtown Eastside of Vancouver found a rapid increase of fentanyl-positive urine samples over a 5-month period in 2017 among a high-risk sample; all participants reporting non-prescription opioid use tested positive for fentanyl by the final month [1]. An unprecedented number of people, more than 1420, died of illicit drug overdoses in British Columbia, Canada, in 2017 and 81% of suspected deaths involved fentanyl [2]. The overdose crisis is not limited to British Columbia as a report by the US National Center for Health Statistics found 42,249 US opioid drug fatalities in 2016 alone [3].

Secondly, illicit drug overdoses were presenting in unfamiliar ways. While opioid overdoses typically feature pinpoint pupils, respiratory depression, and unconsciousness [4], Insite nurses and staff observed atypical overdose presentations including muscle rigidity, particularly stiff posturing, chest wall and jaw rigidity, dyskinesia, low or irregular heart rate, confusion or delirium, and anisocoria or unequal pupils. Dyskinesia reflected a spectrum of involuntary muscle movements ranging from myoclonic jerks and twitches to more severe cases of chorea including uncontrollable flailing of limbs, and rolling around on the floor. Some of these atypical overdose features presented without other typical opioid overdose characteristics, which caused a delay for staff to recognize these events as opioid overdoses.

A review of coroner reports of fentanyl-related deaths led researchers to hypothesize that acute chest wall rigidity was a significant and previously unreported factor leading to increased mortality among illicit opioid users [5]. While largely unknown in association with illicit drug use, fentanyl-induced muscle rigidity is a well-known complication in anesthesia practice first described in 1953 [5–9]. For example, an anesthesiology case study that featured a 60-year-old man who became less responsive, with open eyes and a staring gaze, had a stiff neck and clenched jaw and rigid chest wall immediately following fentanyl administration [6]. High-dose and fast administration, extremes of age, critical illness with neurological or metabolic disease, and concurrent use of substance that modify dopamine levels, such as cocaine, methamphetamine, amphetamine, and other drugs [10] increase the likelihood for the development of opioid-induced rigidity [7, 8]. Consequently, though anesthesia literature did not explore fentanyl use outside of medical settings, people with drug addictions would qualify as high risk. Chest wall rigidity, also termed “wooden chest” was observed to be more frequent with synthetic lipid-soluble compounds such as fentanyl, acetylfentanyl, alfentanil, and sufentanil, and less frequent with other opioids [5–7].

Much less is known about dyskinesia, anisocoria, low or irregular heart rate, and confusion. Descriptions of these other atypical presentations were included in three anesthesia case reports with dyskinesia [11–13] and three that documented anisocoria [14–16]. For example, a case study of a 26-year-old man who was administered fentanyl anesthesia for surgery exhibited similar characteristics as overdose cases from Insite featuring dyskinesia [11]. In four out of five times the young man underwent surgery, doctors noted purposeless, large-amplitude involuntary movement of his upper limbs while the patient was awake. In the single occasion when the patient was not exposed to fentanyl, involuntary movements were not observed [11].

Although a recent case study from Insite has described fentanyl-induced muscle rigidity following intravenous drug use [17], there was little to be found in the community, addictions and harm reduction literature regarding these atypical overdose presentations. This paper reviews overdose presentations at Insite and describes five forms of atypical overdose presentations that may have occurred without other typical opioid overdose characteristics.

## Methods

Insite staff began to observe atypical overdose presentations in substantial numbers starting in October 2016. A descriptive review of clinical records at Insite for October 2016 to April 2017 was undertaken to quantify and describe atypical overdose presentations, defined as those that did not fit the typical presentation of opioid overdoses. Clinical records at Insite are recorded in a database that includes drop-down fields such as substance consumed, overdose (yes/no), overdose features, and overdose interventions including naloxone administration (yes/no), as well as free text fields where details regarding overdose presentation and response can be recorded. Overdoses are identified and managed according to Insite’s clinical protocols which are designed to account for the spectrum of overdose presentations that can arise in the context of polysubstance use. An overdose record is created in the database when staff must initiate an overdose response. Clinical details of the overdose response are charted in the overdose record at the time of the event. Overdose records are then printed and signed by involved staff. Opioid overdose typically includes combinations of the following symptoms: decreased respiratory rate, altered mental status ranging from mild euphoria or lethargy to coma, constricted pupils, decreased bowel sounds, dry skin, and/or low to regular heart rate and blood pressure as the overdose progresses [4, 18–21]. All overdoses in the study period were reviewed for the number of atypical overdose presentations, temporal trends over the study period, concurrent symptoms, and interventions employed by staff. For the purposes of this analysis, charting notes were evaluated for what appeared to be the main atypical presentation characteristics and other concurrent symptoms. Criteria for atypical presentation were developed through consultation with Insite nurses and front line staff. A research consultant with a Masters in Medical Anthropology completed the review in consultation with the Insite Nurse Coordinator. The review began as a quality improvement project for Insite staff to better understand changes in overdose presentation in order to improve the care they were providing to clients. After sharing the findings within the regional health authority, it was recommended that they be shared with the broader addictions and harm reduction community. Ethical considerations were discussed with the UBC Clinical Research Ethics Board, who waived the need for approval as the data was not collected for research purposes.

## Results

Insite staff responded to 1581 overdoses from October 2016 and April 2017 (Table 1). Of the total number of overdoses, 497 (31.4%) included atypical features. While the total number of overdoses per month peaked in December 2016, the proportion of atypical overdose presentations per month appeared to increase over the study period (Table 1). The number of overdose presentations at Insite has been increasing in recent years but this study was conducted during a period of time when British Columbia was experiencing a surge in overdose deaths, which peaked in December 2016, and where in addition to fentanyl, carfentanyl was increasingly detected across the province, which may have influenced these findings [22, 23]. Overdose events have been increasing at Insite with 768 overdose events in 2015 and 1782 in 2016 and 2151 in 2017 [22]. Of the 497 atypical overdose presentation cases, 420 (84.5%) were treated with oxygen and 343 (69.0%) were treated with naloxone. Seventy-five cases (15.1%) were transferred to hospital by ambulance after initial first response by Insite staff. The rate of hospital transfer was higher in the early months when staff may have been less familiar with atypical presentations than in the latter months of the study (i.e., 23.1% in November 2016 vs 11.1% in April 2017). None of these typical or atypical overdoses at Insite resulted in death.

**Table 1:** Overdose presentations at a supervised injection site, Vancouver Canada (2016–17)

Month	Total number of overdoses	Typical overdose presentation	Atypical overdose presentation	Percentage of atypical overdose presentations
October 2016	168	129	39	23.2%
November 2016	319	243	76	23.8%
December 2016	354	261	93	26.3%
January 2017	223	145	78	35.0%
February 2017	176	103	73	41.5%
March 2017	169	101	68	40.2%
April 2017	172	102	70	40.7%
Total	1581	1084	497	31.4%

Of these, 485 fit into five categories of atypical features: muscle rigidity, dyskinesia, slow or irregular heart rate, confusion, and anisocoria (Table 2). Forty-seven cases (9.7%), involved a combination of features. Combinations of muscle rigidity and dyskinesia were the most common with 15 cases and dyskinesia and low heart rate was the second most common with 11 cases. The minority of atypical overdose presentations that did not fit the five categories included allergic reactions, seizures, expressive aphasia, legs giving out, overdose charting where “other” atypical presentation was noted but staff did not elaborate further, and clients reported feeling strange without any other observable atypical overdose features.

**Table 2:** Atypical overdose presentations at a supervised injection site, Vancouver, Canada (2016–17)

Atypical overdose presentation	Number of cases	Percentage of total atypical overdose presentations	Percentage of total overdose presentations
Muscle rigidity	240	48.30%	15.2%
Dyskinesia	150	30.2%	9.5%
Low or irregular heart rate	69	13.9%	4.4%
Confusion or delirium	24	4.80%	1.5%
Anisocoria	2	0.40%	0.1%
Other	12	2.4%	0.8%

Most clients reported using heroin or combinations of heroin and cocaine or methamphetamine in the atypical overdose presentations discussed, with the exception of a minority of cases. These included a few cases attended offsite responded to by non-nursing staff where the drug used was not reported, one case each where the client reported using cocaine or crystal methamphetamine only and eight cases where the client reported using fentanyl. Examples in the descriptions below were with clients who reported using heroin unless otherwise noted.

### Muscle rigidity

Muscle rigidity ranged from jaw clenching to decorticate posturing with arms bent in towards the body, legs held out straight, clenched fists, and overall stiffness. Decorticate posturing raised the concern of Insite staff, since it may be associated with brain injury. Muscle rigidity was the most common type of atypical overdose presentation observed in 48% of atypical cases (Table 2). The most common body part associated with rigidity was the jaw, mentioned in 65 cases (27% of rigidity cases). While jaw clenching may occur in typical opioid overdoses as central nervous system and respiratory depression progresses, it is unusual to occur at or near onset. Nurses expressed an inability to insert airways due to jaw clenching while the client was unresponsive, clenched fists or finger stiffness that interfered with pulse oximeter monitoring, and stiff posturing that interfered with lowering the client to the floor. Body, chest, or torso rigidity or rigid posturing was mentioned in 56 cases (23%). Of these, 13 cases (5%) specifically mentioned chest, torso, or trunk rigidity.

Atypical overdose cases that involved muscle rigidity also often included concurrent features such as pale or cyanotic face (90% of muscle rigidity cases), unresponsive to verbal (81%) or pain (75%) stimuli and breathing stopped (62%). Cases with muscle rigidity were more likely to include concurrent symptoms of unresponsiveness to verbal or pain stimuli, inability to speak (31%), passed out (30%), limp (29%), and cold or clammy skin (13%) than other types of atypical overdose presentations. While rare overall, muscle rigidity was also accompanied by reports of seizure (6% of muscle rigidity cases) and tightness of chest (1%).

There was a range of severity for muscle rigidity cases described. An example of a mild case involved a client slumped over in his booth with his neck hyperextended. Many of the muscle rigidity cases were more severe with clients described as unresponsive and rigid, sometimes with their eyes wide open. An example is a client who presented with a flexed posture, clenched hands, and eyes open but was non-responsive. In another example, a client was found rigid standing against the injection room wall with eyes wide open but non-responsive. The client was unable to communicate and staff were unable to move him or to get him to sit down. Another overdose involved jaw clenching at the onset and intermittent posturing with extension and contraction of the arms while the neck was hyperextended. The jaw clenching resolved with oxygen and naloxone administration. Early in the study period, muscle rigidity was sometimes described as resembling seizure-like activity since staff lacked the language to describe the new phenomenon they were seeing.

### Dyskinesia

Involuntary muscle movements characterize dyskinesia, sometimes called flailing. Chorea, characterized by dance-like flailing of limbs, is frequently reported with cocaine use, though less frequently with opioid use [24]. Opioid use is more frequently associated with myoclonus, sudden muscle contractions that results in jerks or twitches [24]. However, in overdose charting at Insite, large, purposeless movement of limbs, gross uncoordinated flailing, spontaneous movement of limbs, and rolling around on the floor were noted while using opioids. Dyskinesia was the second most common atypical overdose presentation with 150 cases (30% of atypical cases) (Table 2).

Atypical overdose cases that involved dyskinesia also often included concurrent features such as pale or cyanotic face (69% of dyskinesia cases), breathing slowed (51%), and unresponsive to verbal stimuli (52%). While flailing has been strongly associated with stimulant use [24], pallor or cyanosis is not common in stimulant-induced flailing and marked the event as opioid-induced flailing for Insite staff. Cases with dyskinesia were more likely to have sweaty or hot skin (29% of dyskinesia cases), incoherent vocalizations (25%), and face blue, pale, or flushed skin (5%) than other atypical overdose presentations.

An example was a client who presented with dyskinesia and distress, yelling “I don’t understand what is happening to me”. The client was alert, responsive, and not exhibiting other typical features of opioid overdose. Another example was a client who presented first by slamming his arms onto the table while making incoherent sounds then collapsing onto the floor and beginning to flail. Continued dyskinesia, incoherent vocalizations, and an inability to respond to staff direction was interspersed with brief periods of lucidity when the client apologized for his actions with clear speech. In another example of dyskinesia, the client was described as rolling around on the floor with limbs flailing, yelling, and crying incomprehensibly and becoming increasingly sweaty.

Because of the restlessness of dyskinesia, depression of the respiratory system typical of opioid overdoses was harder to identify. Consequently, dyskinesia would sometimes occur before staff realized an overdose was occurring. In one example where the first sign of the overdose reported was dyskinesia and incoherent vocalizations, staff did not realize the client was having an overdose until they saw that the client’s lips were rapidly becoming blue. In another example, a client abruptly punched the injection room booth wall and threw his chair. Insite staff attempted to de-escalate when they realized that the client’s lips were blue and he was having an overdose. After the client was resettled in his booth, he abruptly became non-responsive and began rolling on the floor while making incoherent vocalizations. It became practice at Insite to treat cases of dyskinesia with pale and sweaty skin with naloxone. Even with restlessness, the paleness and pinned pupils suggested an opioid event.

### Low or irregular heart rate

While a low heart rate can be seen in opioid overdoses as depression of the respiratory system progresses and the heart beings to lose compensatory mechanisms, it is atypical at Insite where overdose response is rapid and often at onset of the overdose. Instead, it is typical for the heart rate to increase during the early stages of an overdose as the body compensates for lower respiration. However, during the study period, there were 69 cases (14% of atypical cases) where the heart rate was reported to be lower than 60 beats per minute (bpm) or was irregular (Table 2). For example, a client who reported using fentanyl had an overdose characterized by a slowing of breathing, cyanotic face, and heart rate dropping to 38 bpm.

Atypical overdose cases that involved low or irregular heart rate also often included concurrent features of pale or cyanotic face (86% of low or irregular heart rate cases), slowed breathing (58%), and unresponsiveness to verbal (62%) or pain (52%) stimuli. While rare overall, low or irregular heart rate was also accompanied by vomiting (4% of low or irregular heart rate cases) and chest pain (1%).

### Confusion

Confusion or delirium was noted in 24 overdoses (5% of atypical cases) during the study period. An example includes an overdose where the client was confused and unable to respond verbally to questions. However, unlike typical opioid overdoses where the respiratory system becomes depressed, the client’s oxygen saturation was relatively high at 89%.

In another example, a client reported feeling unwell and exhibited drowsiness, confusion, and a delayed response or failure to answer questions. No other overdose features were noted but the client became increasingly alert after naloxone was administered. The response to the opioid antagonist suggests that the confusion was due to an opioid even though typical opioid overdose symptoms were not observed.

A further example was an overdose where the client exhibited no physiological symptoms of an overdose other than altered level of consciousness. The client exhibited confused facial expressions and was unable to provide basic personal details such as the events leading up to the moment, how she arrived at the site or where she lived.

Atypical overdose cases that involved confusion also often included concurrent symptoms of pale or cyanotic face (71% of confusion cases) and slow breathing (63%). Overdose cases involving confusion were more likely to exhibit slow breathing and present without any other concurrent overdose features (17%) than other types of atypical overdose presentations.

### Anisocoria

While opioid overdoses typically feature pinpoint pupils [4, 18–21], there were two cases of anisocoria, or unequal pupils, during the study period (Table 2). Both cases occurred in January 2017. In one case, the client exhibited confusion and difficulty with word finding. No other features were noted except that the client’s left pupil was 7 mm while his right was 3 mm.

In another case, the client was noted to be unresponsive with a rigid body with flexed arms and wrists, jaw clenched at onset, and a tremor to her limbs. Following naloxone administration, staff noted that her right pupil was dilated while the left pupil was constricted. The client exhibited some disorientation but a neurological exam was otherwise normal.

## Discussion

During the 7-month study period, Insite staff responded to 485 atypical overdose presentations featuring muscle rigidity, dyskinesia, slow or irregular heart rates, confusion, and/or anisocoria out of a total 1581 overdoses. The similarity between the adverse events observed following fentanyl administration in the anesthesiology context and the cases reviewed in this study supports the hypothesis that fentanyl contamination in the illicit drug supply may be responsible for these atypical overdose presentations. More research is needed to further understand this hypothesis, as well as to better understand low or irregular heart rate at overdose onset and confusion, especially without other typical presentations of opioid overdose.

Burns and colleagues theorized that the recent increase in overdose mortality may be due in part to complications of chest wall rigidity associated with intravenous fentanyl use, which includes an abrupt inability to ventilate [5]. Even with emergency response, intubation may be difficult due to clenched jaw and rescue bag-valve-mask ventilation may prove ineffective [6, 7, 25–27]. Likewise, Insite staff described difficulties in inserting airways with a clenched jaw, ventilating during chest rigidity, and measuring oxygen saturation with stiff fingers and clenched hands. Numerous anesthesia reports [5, 6, 26–29] as well as a recent British Columbia guideline for managing fentanyl-induced muscle rigidity in the community [30] recommended treating chest wall rigidity with rapid administration of naloxone.

Additionally, Insite staff described challenges in recognizing an overdose was occurring with presentations such as dyskinesia or confusion in the absence of other typical opioid overdose symptoms. In contrast to typical opioid overdose presentations, there were clients who were conscious and awake, yet seemed to be experiencing an overdose. It is important to recognize that presentations involving muscle rigidity, dyskinesia, low or irregular heart rates, anisocoria, and confusion/delirium could be opioid overdoses and require treatment with oxygen and naloxone. When Insite staff were beginning to observe an increasing number of atypical overdose presentations, there was gap in understanding what was happening. Existing understanding of opioid overdose and tools available were insufficient in explaining the shift in overdose presentations that staff were witnessing. This was reflected in the overdose charting as there were many different ways of describing atypical overdose presentations and even question marks written into the charts at the beginning. However, as frontline staff experienced atypical overdose presentations more often, descriptions became more standardized. Additionally, learning from experience was evident in the interventions employed. Staff often called paramedics in the early months, but by the end, staff were more confidently treating clients with atypical overdose presentations with naloxone and oxygen.

This change in overdose presentations is occurring at a time when harm reduction services are rapidly evolving in response to the overdose crisis. In addition to sites like Insite where nurses supervise consumption, overdose prevention sites now exist where people with lived experience (peers) who have been trained in overdose response perform supervision. This emphasizes the importance of sharing information, especially with local communities where peer-run consumption sites are located. The findings of this paper will also be used to create a community knowledge translation resource.

Additionally, the learning experiences related to atypical overdose presentations at Insite highlight the importance of sharing information between supervised consumption sites, overdose prevention sites, and other community partners involved in harm reduction and overdose response. In addition to the immediate benefits of supervised consumption sites in terms of preventing overdose deaths, clinical observations from supervised consumption sites can contribute to the community as they allow insights into processes and events that are usually unseen. These insights may assist with more effective management of overdoses. Supervised consumption sites are innovative spaces for community-based research and knowledge translation.

This study was designed as a quality improvement project as opposed to a research study, which might have employed different methodologies. The study is limited as a descriptive study with no control group, self-report of the drugs consumed, and biases due to different reporting by different writers and brief reporting during days with high numbers of overdoses, especially in November and December 2016. Though most of the overdoses in this study occurred inside the facility, there was a small minority of overdoses where staff responded outside the facility. The descriptions of overdose characteristics at onset were less accurate for interventions outside of the facility’s injection room. For offsite overdoses, atypical presentations may be under-reported as community bystanders sometimes already administered naloxone before Insite staff arrived at the scene. Additionally, at the early stage, there may have been underreporting by staff at Insite due to lack of familiarity with these overdose presentations. Consequently, because of these limitations, the number of atypical overdose presentations reported here can be considered underestimates.

## Conclusion

It is increasingly recognized that an unregulated drug supply is vulnerable to contamination. As the drug supply is increasingly contaminated with fentanyl and other synthetic opioids, overdoses may present with atypical features with or without other typical opioid overdose characteristics. It is important to recognize that muscle rigidity, dyskinesia, slow or irregular heart rates, confusion, and anisocoria may be observed as part of overdose presentations and should still be treated with naloxone and oxygen.

The severity and persistence of the overdose crisis calls for more research grounded in the experiences of people who use drugs and the frontline workers who are able to observe overdose presentations from their onset. Observations and insight from supervised consumption sites may be valuable resources to further understanding and management of overdoses.
